# Ascorbic acid 6-palmitate modulates microglia M1/M2 polarization in lipopolysaccharide-stimulated BV-2 cells via PERK/elF2α mediated endoplasmic reticulum stress

**DOI:** 10.1186/s12906-022-03780-1

**Published:** 2022-11-18

**Authors:** Qian Li, Yao Wu, Xue-shen Chen, Tao Zeng, Lin-ling Liu, Zi-qi Feng, Dan-yang Liu, Ling Zhu, Li-hong Wan

**Affiliations:** 1 Grade 2018, West China School of Basic Medical Sciences and Forensic Medicine, Sichuan University, Sichuan 610041 Chengdu, PR China; 2 Department of Pharmacology, West China School of Basic Medical Sciences and Forensic Medicine, Sichuan University, 3-17 Renmin South Road, Sichuan 610041 Chengdu, PR China; 3 NHC Key Laboratory of Chronobiology (Sichuan University), West China School of Basic Medical Sciences and Forensic Medicine, West China Second University Hospital, Sichuan University, 3-17 Renmin South Road, Sichuan 610041 Chengdu, PR China; 4 Grade 2019, West China School of Basic Medical Sciences & Forensic Medicine, Sichuan University, Sichuan 610041 Chengdu, PR China

**Keywords:** Ascorbic acid 6-palmitate (L-AP), M1/M2 polarization, ER stress, PERK/elF2α

## Abstract

**Background:**

Neuroinflammation-mediated microglia polarization is a major process in various central nervous system (CNS) diseases. Endoplasmic reticulum (ER) stress contributes to the inflammatory signals as well as to microglia polarization in lipopolysaccharide (LPS) induced neuroinflammation. Ascorbic acid 6-palmitate (L-AP) has been broadly used as a dietary antioxidant in foods and demonstrated a strong inhibitory effect on 5-LOX; however, the specific anti-inflammation mechanisms remain unclear. In this study, we investigated the effects and possible mechanisms of L-AP on LPS-induced neuroinflammation in BV-2 cells.

**Methods:**

Immortalized murine microglia cell line BV-2 cells were employed to assess the effect of L-AP to modulate microglia M1/M2 polarization in vivo, and the molecular mechanism was evaluated by qRT-PCR and Western blotting analysis. Molecular docking was used to predict the binding activity of L-AP with protein kinase R-like ER kinase (PERK).

**Results:**

L-AP at 62.5 µM significantly modulated LPS-induced microglia M1/M2 polarization (increases of interleukin (IL)-10 and arginase-1 (Arg-1) transcriptions) independent of cell growth. Besides, L-AP at 62.5 µM significantly down-regulated glucose-regulated protein 78 (GRP78) and CCAAT/enhancer-binding homologous protein (CHOP) mRNA levels. Similar data were shown in the tunicamycin (TM) induced ER stress cells model. Moreover, the protective effect of L-AP on TM-induced microglia M1/M2 polarization was similar to that of 4-phenyl butyric acid (4-PBA), the ER stress inhibitor. Molecular docking results indicated L-AP might directly bind with PERK, with a binding affinity of -7.7 kcal/mol. A further study unveiled that L-AP notably inhibited LPS-induced PERK/ eukaryotic initiation factor 2α (elf2α) activation.

**Conclusion:**

Together, this study revealed that L-AP possessed its effect on the reconstruction of microglia M1/M2 polarization balance in LPS-stimulated BV-2 cells via modulating PERK/elF2α mediated ER stress.

**Supplementary Information:**

The online version contains supplementary material available at 10.1186/s12906-022-03780-1.

## Introduction

The neuroinflammatory response is a defense mechanism to protect the central nervous system (CNS) against various invading pathogens or injury-related products. However, uncontrolled neuroinflammatory response interferes the homeostatic integrity and plays a critical role in multiple neurological and neurodegenerative disorders through redundant production of cytokines. Microglia are the resident mononuclear phagocytes of the CNS, which display apparent duality in response to inflammatory injury and repair including neurotoxic M1-type and neuroprotective M2-type, which is called heterogeneity [1]. On physiological conditions, neurotoxic M1-type and neuroprotective M2-type maintain balance. However, on the condition of neuroinflammation, activated microglia tends to be M1-type, which expresses or releases amounts of proinflammatory cytokines, such as inducible nitric oxide synthase (iNOS), tumor necrosis factor (TNF)-α, and interleukin (IL)-1β. Due to the lack of neuroprotective M2-type, the secretion of anti-inflammatory cytokines including arginase-1 (Arg1) or IL-10 and trophic factors is not enough to resolve local inflammation. Thus, the balance of M1/M2 polarization tends to be M1 and further aggravates brain injury by mediating uncontrolled neuroinflammation [2, 3]. Therefore, reconstruction of M1/M2 polarization balance has been widely accepted as a potential therapeutic approach in multiple neuroinflammatory diseases, especially in sepsis [4].

Endoplasmic reticulum (ER) stress is a coordinated adaptive process initiated by three ER-localized transmembrane sensors including the inositol-requiring enzyme-1 (IRE-1), protein kinase receptor-like ER kinase (PERK), and activating transcription factor 6 (ATF6). Accumulating evidence indicates that sustained ER stress contributes to neuronal injury owing to causing abnormal inflammatory signals cascades [5], especially in lipopolysaccharide (LPS) induced neuroinflammation [6]. Importantly, recent research has demonstrated the potential mechanism of ER stress involved in neuroinflammation is activating microglia [7]. Also, inhibition of ER stresses significantly protected against neuroinflammation [8]. However, there are still some different views on the role of ER stress degree in neuron damage. A recent study showed that maintaining ER stress at a moderate level inhibits neuronal death in mice, which suggested the role of ER stress in neuroinflammation-related disease is context-specific and complex [9]. Altogether, ER stress exerts dual functions in response to neuroinflammation via regulating microglia polarization phenotype. Precise modulation of ER stress might be a potent therapeutic target for various neuroinflammatory diseases.

**Fig. 1:**
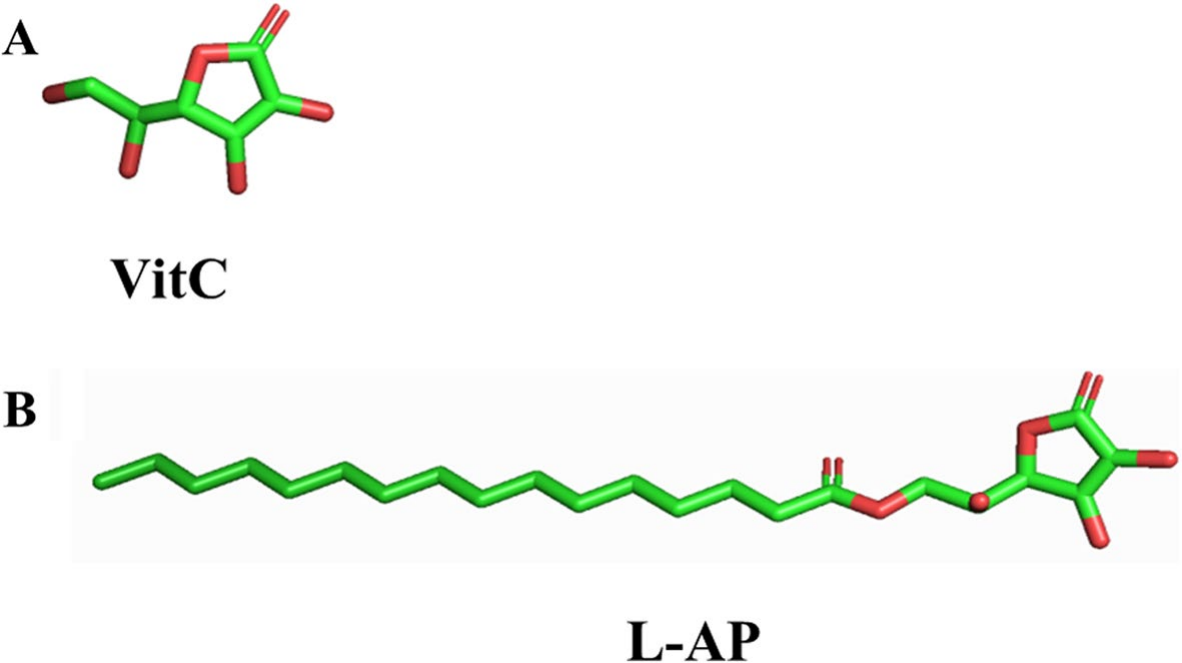
Chemical structures of VitC and L-AP. **A** Chemical structures of VitC; **B** Chemical structures of L-AP

Ascorbic acid 6-palmitate (L-AP) is a derivative of ascorbic acid (Fig. 1A), which is different from most antioxidant esters. The esterification occurs at C6, instead of esterifying the active-center hydroxyl group at C3,4, which preserved the activity of the dienediol structure of VitC and increased its stability [10]. And it’s absorbable and durable due to its higher lipid-soluble. It has been broadly used as a dietary antioxidant in food additives or dermatological products [11]. More importantly, the binding affinity of L-AP with 5-lipoxygenase (5-LOX), the key enzyme in pro-inflammatory leukotriene (LT) formation, is significantly improved via its long hydrophobic alkyl chain (Fig. 1B). Thus, L-AP could more effectively inhibit 5-LOX activity than ascorbic acid [12]. All evidence has indicated the potential anti-inflammatory effect of L-AP. However, no study has been carried out concerning the modulation of L-AP on microglia M1/M2 polarization to our knowledge.

Based on the literature evidence, we designed our study to investigate whether L-AP possessed its effect on the reconstruction of microglia M1/M2 polarization balance in LPS-stimulated BV-2 cells via modulating endoplasmic reticulum stress.

## Materials and methods

### Cell Culture and Treatment

Immortalized murine microglia cell line BV-2 cells (Procell Life Science & Technology Co., Ltd, China) were cultured in Dulbecco’s modified Eagle’s medium (DMEM) with 10% Fetal Bovine Serum (FBS) and 1% penicillin/streptomycin at 37°C in humidified 5% CO_2_ and 95% air. To establish the BV-2 cells M1 polarization model, BV-2 cells were incubated with LPS (1 µg/mL; Sigma-Aldrich, USA) for 24 h. To confirm the role of ER stress on BV-2 cells’ M1 polarization, cells were incubated with tunicamycin (TM; Sigma-Aldrich, USA) 6 nM or 4-phenyl butyric acid (4-PBA; Sigma-Aldrich, USA) 30 µM for 24 h.

### Cytotoxicity assay

50 mg L-AP powder was dissolved in 1 mL of dimethylsulfoxide (DMSO) to prepare a 125 mM L-AP storage solution. Cells were plated into 96-well plates at the density of 1×10^3^ / well and incubated with different concentrations of ascorbic acid 6-palmitate (0, 2, 4, 8, 16, 32, 62.5, 125, 250, 500 µM) for 24 h. Then, 10 µL of cell counting kit-8 (CCK-8) reagent (Dojindo, Japan) was added to each well and incubated at 37°C for 4 h. After incubation, OD values were measured at 450 nm using an Epoch microplate spectrophotometer (Bio-Tek, USA).

### Enzyme-linked immunosorbent assay (ELISA)

Cells were plated into 6-well plates at the density of 1 × 10^6^ / well. After treatment, the concentrations of interleukin-6 (IL-6) and TNF-α in cell supernatant were measured using a microplate reader (Bio-tek Epoch) at 450 nm with ELISA kits (Multisciences Biotech, Co., Ltd, Hangzhou, China) according to the manufacturer’s instructions. The absorbance of the reaction products was calibrated at 570 nm.

### Real-time polymerase chain reaction (qRT-PCR)

Total RNA was extracted with Trizol reagent and reverse-transcribed using the Revert Aid First Strand cDNA Synthesis Kit (Thermo Fisher Scientific, USA). Real-time PCR was performed using qTOWER3G (Analytik Jena, Germany). PCR amplification conditions involved the initial denaturation for 2 min at 95°C and 40 cycles of 15 s at 95°C and 60 s at 60°C. PCR primer sequences were as follows (forward primer and reverse primer, respectively): *gapdh* (122 bp): 5′-AGGTCGGTGTGAACGGATTTG-3′, 5′-TGTAGACCATGTAGTTGAGGTCA-3′; *inos* (118 bp): 5′-ACGAGACGGATAGGCAGAGA-3′, 5′-CACATGCAAGGAAGGGAACT-3′; *il-10* (100 bp): 5′-CTTACTGACTGGCATGAGGATCA-3′, 5′-GCAGCTCTAGGAGCATGTGG-3′; *arg-1* (118 bp): 5′-GACCTGGCCTTTGTTGATGT-3′, 5′-CCATTCTTCTGGACCTCTGC-3′; *chop* (382 bp): 5′-TGTTGAAGATGAGCGGGTGG-3′, 5′-GATTCTTCCTCTTCGTTTCCTGG-3′; *grp78* (117 bp): 5′-TTCTCAGCATCAAGCAAGGA-3′, 5′-CATGGTAGAGCGGAACAGGT-3′. The data were analyzed with LightCycler®96 software (Roche Diagnostics, Germany).

### Protein preparation and Western blotting analysis

Total proteins were extracted using RIPA Lysis Buffer (Beyotime Institute of Biotechnology, China) and the protein concentrations were measured with a BCA protein assay kit (Beyotime Institute of Biotechnology, China). Equal amounts of total protein (40 µg/10 µL) were loaded onto 8% SDS PAGE gels at 120 v for 60 min, electrotransferred to PVDF membranes, cropped the PVDF membranes according to the position of the marker, blocked in 5% no-fat milk at room temperature for 2 h, and incubated with the diluted primary antibodies (Table 1) at 4 °C overnight in turn. After washing three times with TBST, the membranes were probed with horseradish peroxidase-conjugated secondary antibody at room temperature for 2 h. β-Actin was the loading control. The protein was detected by chemiluminescence reagent (GE Healthcare) and the protein band density was quantified using Image J software.

**Table 1:** Antibodies

Name	Experiment	Vendor	Dilution
Mouse Monoclonal anti-β-Actin antibody	WB	CST, USA	1:1000
Rabbit Monoclonal anti-PERK antibody	WB	CST, USA	1:1000
Rabbit Monoclonal anti-p-PERK antibody	WB	SAB, USA	1:600
Rabbit Monoclonal anti-p-eIF2α antibody	WB	CST, USA	1:1000
Goat anti-Mouse IgG Secondary antibody	WB	SAB, USA	1:5000
Goat anti-Rabbit IgG Secondary antibody	WB	SAB, USA	1:5000

### Molecular docking

Molecular docking studies were performed using Accelrys Discovery Studio 4.0 (DS, BIOVIA Software, Inc., USA) to gain insights into the plausible binding modes of L-AP and PERK. The crystal structure of PERK (PDB bound to GSK6924) was obtained from the Protein Data Bank (http://www.rcsb.org/). The molecular structure of L-AP was pre-treated by DS and defined as the ligand. Also, the protein molecule of PERK was pre-treated by DS to remove water molecules and original ligands and defined as the receptor. The binding sites of L-AP and PERK were specified by the center of the co-crystallized ligand GSK6924. Then, the pre-processed receptor and ligand were batch-processed using MGLTooLs-1.5.6 to generate files in pdbqt format. Finally, molecular docking of the ligand to the receptor was performed with AutoDock Vina. Other parameters were set as default. Various binding modes were evaluated based on the docking energy, and the score was obtained. Reasonable docking results were selected according to the lowest score. In general, if the binding energy is less than − 1.2 kcal/mol, the docking result is feasible.

### Statistical analysis

All values were expressed as mean ± SEM and statistically analyzed by one-way analysis of variance (ANOVA) with Bonferroni correction (GraphPad Prism version 5). *P* < 0.05 was considered as a statistically significant difference.

## Results

### Ascorbic acid 6-palmitate (L-AP) modulates microglia M1/M2 polarization in LPS-stimulated BV-2 cells

**Fig. 2:**
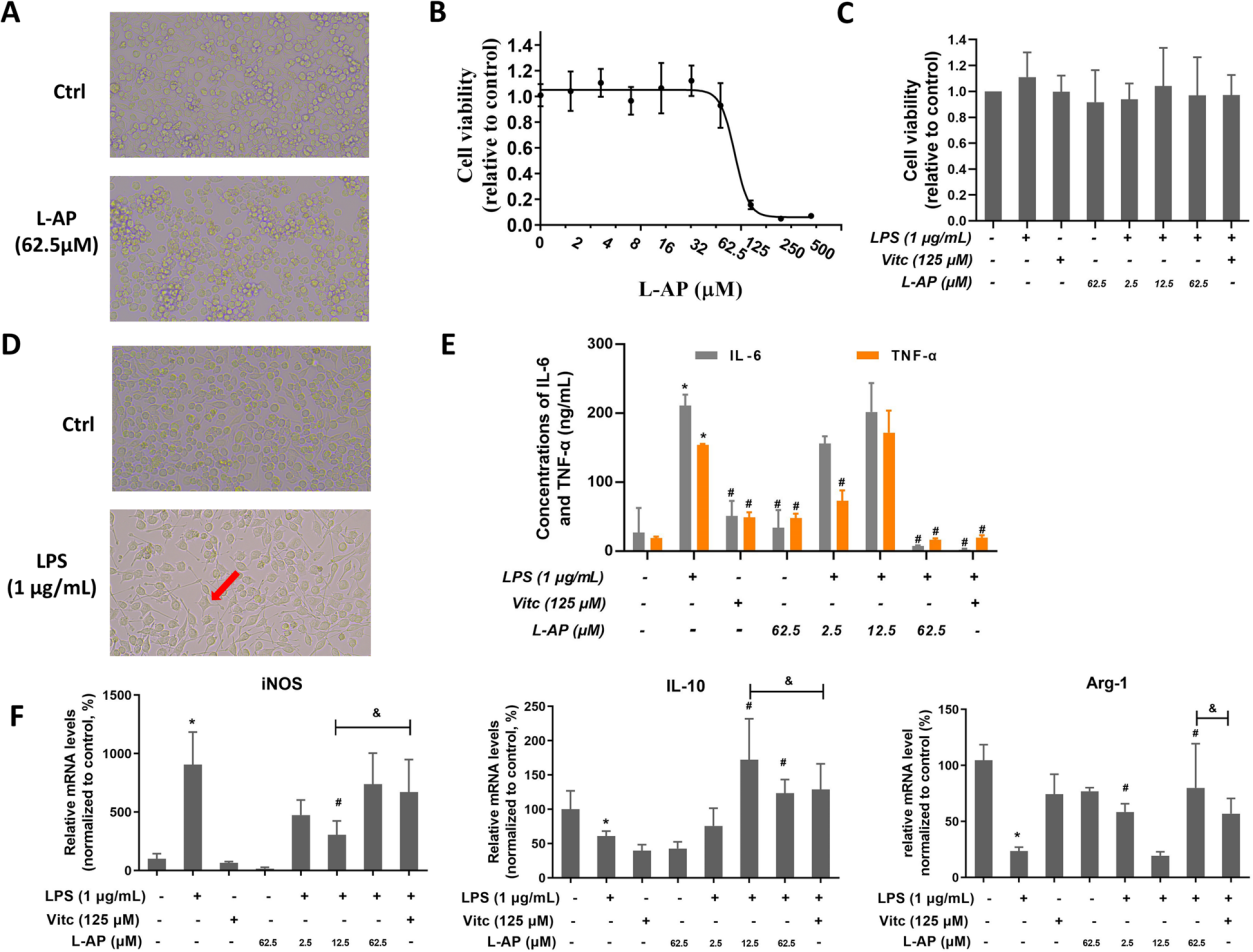
Effects of L-AP pre-treatment on LPS-induced neuroinflammation in BV-2 cells. **A** Bright-field micrographs showing BV-2 morphology (×250); **B**, **C** Effect of L-AP on cell viability in the absence or presence of LPS stimulation in BV-2 cells by CCK8 assay; **D** Bright-field micrographs showing the effect of LPS on BV-2 morphology, the red arrow indicates activated BV-2 cell (×250); **E** The protein concentrations of IL-6 and TNF-α were assayed by ELISA; **F** mRNA levels of *inos*, *il-10* and *arg-1* in BV-2 cells. Values are presented as mean ± S.E.M. ^*^p < 0.05, versus ctrl group (*n* = 5). ^#^*p* < 0.05, versus LPS alone treatment group (*n* = 5). ^&^*p* < 0.05, versus VitC group (*n* = 5)

Ascorbic acid 6-palmitate (L-AP) is a lipid-soluble derivative of ascorbic acid (Vitamin C, VitC), which has been indicated as an antioxidant [10, 11]. To confirm the roles of L-AP on lipopolysaccharide (LPS)-induced microglia activation in BV-2 cells independent of cell growth inhibition, we first examined the potential cytotoxicity of L-AP on BV-2 cells in the absence or presence of LPS stimulation for 24 h by CCK8 assay. After 24 h of exposure to the various concentrations of L-AP, the viability of BV-2 cells did not suffer from significant inhibition at the concentrations below 62.5 µM (Fig. 2A). IC_50_ value of L-AP was 84.56 µM (Fig. 2B). Therefore, concentrations of 2.5, 12.5 and 62.5 µM L-AP were chosen for subsequent experiments. According to our preliminary study, LPS (1 µg/mL) exposure for 24 h did not significantly change cell growth. And as we expected, after 4 h of pre-treatment with different concentrations of L-AP (2.5,12.5 and 62.5 µM), the viability of BV-2 cells was not significantly inhibited by L-AP at all concentrations in the presence of LPS (1 µg/mL) (*P* > 0.05, Fig. 2C). Then, the cellular morphology was observed as an indicator of the microglia activation level in the presence of LPS (1 µg/mL) for 24 h. We found that in the presence of LPS (1 µg/mL), BV-2 cells become spindle-shaped, while in the absence of LPS (1 µg/mL), BV-2 cells are round-shaped (Fig. 2D). Moreover, the ELISA data showed that the concentrations of IL-6 and TNF-α were significantly increased in the presence of LPS (1 µg/mL) for 24 h, while pre-treated with L-AP at 62.5 µM notably prevented LPS-induced IL-6 and TNF-α secretion (*P* < 0.05, Fig. 2E). Subsequently, whether L-AP treatment modulates LPS induced microglia M1/M2 polarization, mRNA levels of M1 marker (*inos*) and M2 marker (*il-10* and *arg-1*) were measured by qRT-PCR. Our data showed that in the presence of LPS (1 µg/mL) for 24 h, the mRNA level of *inos* was significantly increased, and the mRNA levels of *il-10* and *arg-1* were significantly decreased, whereas the M2 marker (*il-10* and *arg-1*) levels were significantly increased by L-AP pre-treatment at 62.5 µM (*P* < 0.05, Fig. 2F). However, L-AP treatment at 62.5 µM did not demonstrate significant inhibition on M1 marker (*inos*) transcription (*P* > 0.05, Fig. 2F). At the same time, although VitC pre-treatment tended to reduce *inos* level and increase the level of M2 markers (*il-10* and *arg-1*) in the presence of LPS (1 µg/mL), there was no significant difference (*P* > 0.05, Fig. 2F). Taken together, microglia were activated and polarized to the M1 phenotype after LPS exposure and the treatment with L-AP at 62.5µM promoted the polarization of microglia to the alternative M2 phenotype, whereas VitC did not switch microglia M1/M2 polarization in this condition.

### Ascorbic acid 6-palmitate (L-AP) switches microglia M1/M2 polarization via alleviating ER stress

**Fig. 3:**
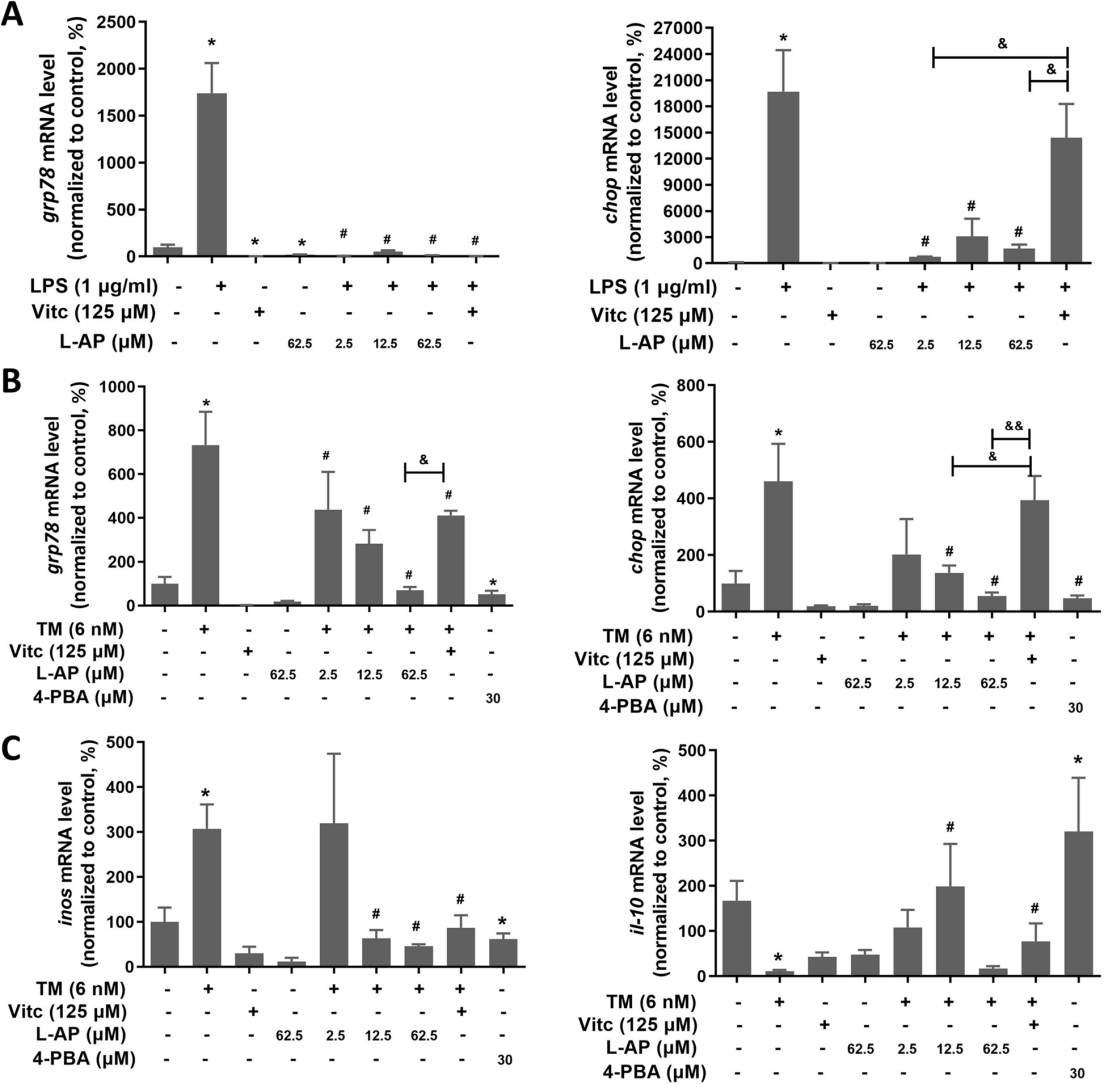
Effects of L-AP pre-treatment on LPS or TM-induced microglia polarization and ER stress in BV-2 cells. **A** mRNA levels of grp78 and chop in LPS-stimulated BV-2 cells; **B** mRNA levels of grp78 and chop in TM-stimulated BV-2 cells; **C** mRNA levels of *inos* and *il-10* in TM-stimulated BV-2 cells. Values are presented as mean ± S.E.M. ^*^p < 0.05, versus ctrl group (*n* = 5). ^#^*p* < 0.05, versus LPS or TM alone treatment group (*n* = 5). ^&^*p* < 0.05 and ^&&^*p* < 0.01, versus VitC group (*n* = 5)

Recently endoplasmic reticulum (ER) stress involved in neuroinflammation and microglial activation has been widely discussed [9, 13]. First, to elucidate the roles of L-AP on LPS-induced ER stress in BV-2 cells, mRNA levels of ER stress markers (*grp78* and *chop*) were measured using qRT-PCR. As shown in Fig. 3A, LPS exposure significantly increased *grp78* and *chop* mRNA levels (*P* < 0.05), and L-AP treatment at all concentrations significantly reversed LPS (1 µg/mL) for 24 h induced increase of *grp78* and *chop* mRNA levels (*P* < 0.05, Fig. 3A). Next, to determine whether ER stress contributes to the protective effects of L-AP on switching LPS induced microglia toward M1 polarization, 6 nM tunicamycin (TM) was used to induce microglia ER stress in BV-2 cells and 30 µM 4-PBA was used to inhibit ER stress according to our preliminary study (Supplementary Fig. 1). As predicted, BV-2 cells’ exposure to TM (6 nM) for 24 h significantly induced ER stress (an increase of *grp78* and *chop* mRNA level) (*P* < 0.05, Fig. 3B) paralleled to M1 polarization of microglia (up-regulation of *inos* mRNA and down-regulation of *il-10* mRNA) (*P* < 0.05, Fig. 3C), whereas L-AP treatment at 62.5 µM notably attenuated TM-induced ER stress and M1 polarization (*P* < 0.05, Fig. 3B C). Especially in alleviating ER stress, L-AP demonstrated stronger effects than VitC (*P* < 0.05, Fig. 3B). In contrast, 30 µM 4-PBA (ER stress inhibitor) treated BV-2 cells demonstrated similar results to L-AP (Fig. 3B C, *P* < 0.05). All these results indicated that L-AP switched microglia M1/M2 polarization via alleviating ER stress.

### Ascorbic acid 6-palmitate suppressed LPS-induced PERK/ eukaryotic initiation factor 2α (elf2α) activation

**Fig. 4:**
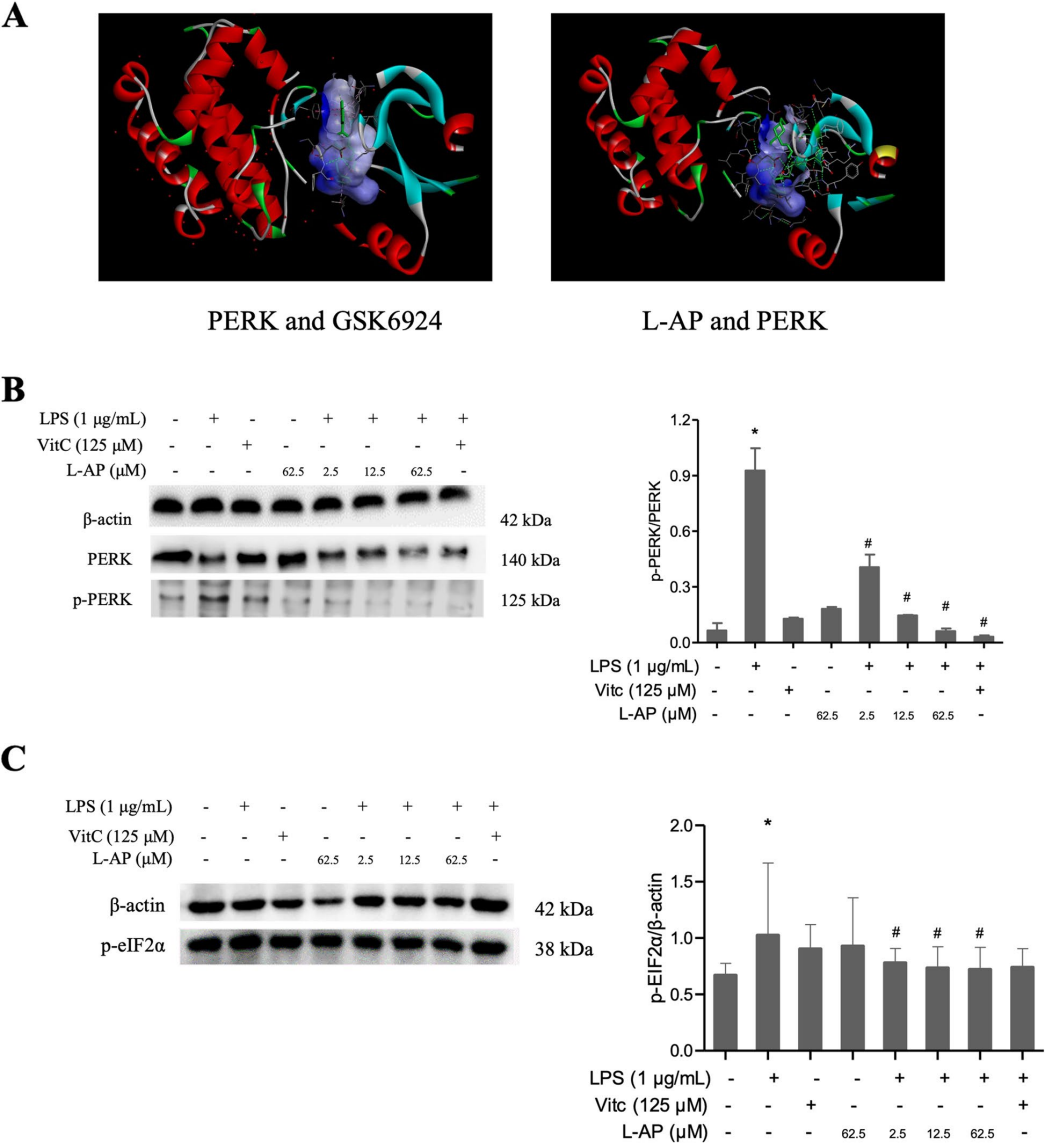
Effects of L-AP pre-treatment on LPS-induced PERK/elF2α activation in BV-2 cells

Protein kinase RNA-like ER kinase (PERK) is one of the transmembrane stress sensors of the unfolded protein response (UPR). After PERK dissociation with GRP78, PERK/eIF2α/CHOP pathway was activated under ER stress conditions [14]. To predict and determine whether L-AP interacts directly with PERK, molecular docking studies were performed. Molecular docking results indicated L-AP might directly bind with PERK, with a binding affinity of -7.7 kcal/mol (Fig. 4 A). Then, to elucidate the role of L-AP on PERK/eIF2α pathway, expressions of PERK, p-PERK and p-eIF2α proteins were analyzed by western blotting. As shown in Fig. 4B C, LPS exposure significantly increased the p-PERK/PERK ratio and p-EIF2α expressions (*P* < 0.05), and L-AP significantly decreased the p-PERK/PERK ratio in a dose-dependent manner and down-regulated the p-eIF2α level in BV2 cells (*P* < 0.05). However, L-AP might act through multiple pathways in regulating microglia polarization, so the effect of LAP on polarization is greater than that of VitC, but the effect on the p-PERK/PERK ratio is less pronounced than that of VitC. These results indicated that L-AP might directly bind with PERK and suppressed LPS-induced PERK/elF2α activation.

## Discussion

Neuroinflammation is widely considered as the common pathological basis of various neurological diseases, including Alzheimer’s disease, Parkinson’s disease, ischemic stroke, traumatic brain injury, and sepsis-associated encephalopathy. Microglia as the first response to injury stimulation are highly plasticity to monitor the brain microenvironment with different polarization phenotypes (M1 or M2) and play critical roles in mediating neuroinflammation via interaction with neurons or astrocytes [15]. Excess-activated microglia (especially the M1 phenotype) release a large number of pro-inflammatory mediators to mediate neurotoxicity by inducing neuron apoptosis, and even directly influencing cognitive function [16]. Hence, inhibition of microglia-related neuroinflammation could promote cognitive function recovery in neurological disorders.

As a fat-soluble derivative of VitC, L-AP exhibits stronger antioxidant activity due to its palmitate side chain, which is attached to the ascorbate ring of VitC [10]. Previous studies have demonstrated that L-AP is easy to be absorbed into cell membranes and operates an anti-inflammatory effect as a strong inhibitor of 5-LOX in the cytoplasm [11]. In the present study, the effect of L-AP on reconstructing microglia M1/M2 polarization balances with LPS-stimulated BV-2 cells was evaluated. The LPS-induced microglia polarization cell model was established at the concentration of 1 µg/mL for 24 h. On this condition, our assessment clearly proposed that 1 µg/mL LPS exposure significantly activated microglia (promoted M1 polarization) independent of cell growth. Moreover, L-AP at 62.5 µM switched the pro-inflammatory M1 phenotype to the anti-inflammatory M2 phenotype, which was characteristic with *il-10* and *arg-1* transcriptions significantly increased. Interestingly, VitC did not demonstrate a modulatory role in microglia polarization phenotype.

Several lines of evidence demonstrated the links between ER stress, neuroinflammation, and microglial activation [9, 13]. A previous study showed that the induction of CCAAT/enhancer-binding homologous protein (CHOP) by TM (the ER stress inducer) was more quickly than that induced by LPS in BV-2 cells, implying the role of ER stress in mediating endotoxin-induced neuroinflammation [17]. In the present study, we found similar effects of TM and LPS exposure in increasing both the grp78 and chop mRNA levels and inducing microglia M1 polarization. However, inhibiting ER stress by 4-PBA (ER stress inhibitor) demonstrated contrary results in BV-2 cells that M1 polarization was significantly suppressed but M2 polarization was notably increased. Combined with previous reports, our results suggested that ER stress may be the primary mode to mediate microglial activation in LPS-induced neuroinflammation. Importantly, different ER stress status has been reported to contribute to the modulation of microglia polarization phenotype [9], such as mild ER stress being beneficial to alleviate neuroinflammation [8] via shifting the microglia phenotype from M1to M2 [9]. Our data indicated L-AP at 62.5 µM moderately attenuated TM-induced ER stress by down-regulation of ER chaperones, such as *grp78* and UPR up-regulates genes such as *chop*, which is beneficial to the inhibition of TM-induced M1 microglia activation by down-regulating *inos* mRNA level. Although the M2 microglia marker *il-10* transcription did not promote by L-AP at 62.5 µM, we found 12.5 µM L-AP could significantly up-regulate *il-10* transcription level. Therefore, this modulatory role of L-AP on microglia plasticity was considered as the reconstruction of microglia M1/M2 polarization balances. On the contrary, VitC did not possess a role in the reconstruction of microglia M1/M2 polarization balance under ER stress-mediated neuroinflammation. Lack of the palmitate side chain may affect the ability of VitC to be transported into the cytoplasm [10].

Under ER stress, three transmembrane stress sensors of the UPR such as PERK, inositol Requiring Enzyme 1 (IRE1), and ATF6 dissociated from GRP78 to initiate various signal pathways to ameliorate ER stress. Among them, the PERK signal pathway is the preferentially activated signal elicited by ER stress [18]. In response to ER stress, PERK autophosphorylated and further phosphorylated the eukaryotic translation initiation factor 2α (eIF2α) to attenuate protein translation globally via downstream transcription factor CHOP [19]. Moreover, inhibition of PERK just attenuated microglia M1phenotype but did not disrupt normal cytokine signaling in microglia [20]. Interestingly, based on our molecular docking prediction, we found PERK might directly bind with L-AP. Subsequently, western blotting results confirmed our prediction that L-AP decreased the p-PERK/PERK ratio in a dose-dependent manner and down-regulated the p-EIF2α level enhanced by LPS in BV2 cells.

## Conclusion

**Fig. 5:**
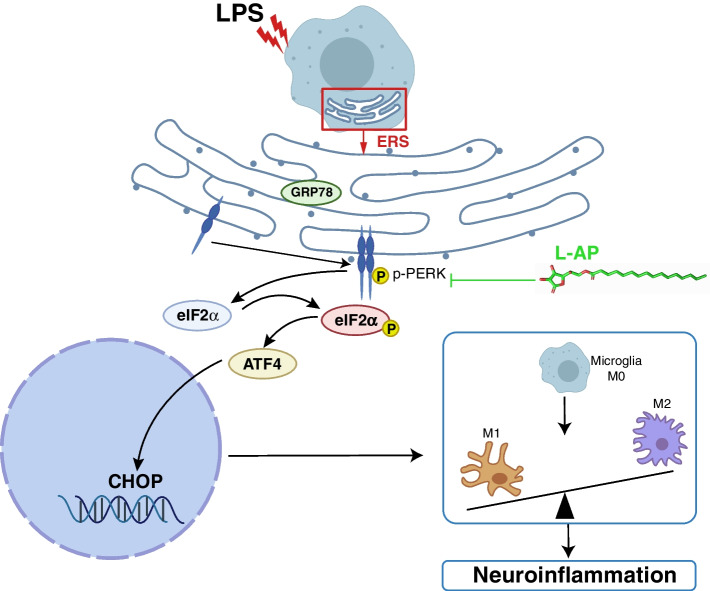
Graphical summary of the results. On exposure to LPS, BV-2 cells were activated toward M1 polarization phenotype through GRP78/PERK/elF2α/CHOP mediated severe ER stress. L-AP as a potential PERK inhibitor possessed its effect on the reconstruction of microglia M1/M2 polarization balance in LPS-stimulated BV-2 cells via modulating PERK/elF2α mediated ER stress

In summary, L-AP as a potential PERK inhibitor possessed its effect on the reconstruction of microglia M1/M2 polarization balance in LPS-stimulated BV-2 cells via modulating PERK/elF2α mediated ER stress (Fig. 5). These results provide the potential references for the future development of drugs based on the reconstruction of microglia M1/M2 polarization balance for the prevention of neuroinflammatory diseases such as sepsis-associated encephalopathy.

(A) Molecular docking between L-AP and PERK; GSK6924, a potent and selective inhibitor of PERK, was used as the positive control; (B) The protein expressions of PERK, p-PERK were analyzed by western blotting, β-actin was used as an internal standard (left panel); The relative levels of PERK, p-PERK (right panel); Full-length blots/gels were presented in Supplementary Fig. 2; (C) The protein expressions of p-EIF2 was analyzed by western blotting, β-actin was used as an internal standard (left panel); The relative levels of p-EIF2α (right panel); Full-length blots/gels were presented in Supplementary Fig. 3. The results were expressed as the ratio of the investigated gene to β-actin. Values are presented as mean ± S.E.M. ^*^p < 0.05, versus ctrl group (*n* = 4). ^#^*p* < 0.05, versus LPS alone treatment group (*n* = 4).

## Supplementary Information

**Additional file 1:** **Supplementary figure 1.** **A** mRNA levels of *grp78* and *chop* in TM-stimulated BV-2 cells; **B** mRNA levels of *inos* and *il-10* in TM-stimulated BV-2 cells; **C** mRNA levels of *grp78* and *chop* in 4-PBA-stimulated BV-2 cells. **B** mRNA levels of *inos* and *il-10* 4-PBA-stimulated BV-2 cells. **Supplementary figure 2.** **A** Full-length blots/gels of β-actin; **B** Full-length blots/gels of PERK; **C** Full-length blots/gels p-PREK. All of them were repeated three times. **Supplementary figure 3.** Images of Full- length blots/gels of β-actin, p-PERK, and PERK, whose membrane edges can be seen. **Supplementary figure 4.** A Full-length blots/gels p-EIF2α; **B **Full-length blots/gels of β-actin. All of them were repeated three times.

## Data Availability

The data and material are available from the corresponding author upon reasonable request.

## Abbreviations

CNS: Central nervous system
iNOS: Inducible nitric oxide synthase
TNF-α: Tumour necrosis factor-α
IL-1β: Interleukin-1β
Arg1: Arginase-1
ER: Endoplasmic reticulum
IRE-1: Inositol-requiring enzyme-1
PERK: Protein kinase receptor-like ER kinase
ATF6: Activating transcription factor 6
LPS: Lipopolysaccharide
L-AP: Ascorbic acid 6-palmitate
5-LOX: 5-lipoxygenase
LT: Leukotriene
DMEM: Dulbecco’s modified Eagle’s medium
FBS: Fetal Bovine Serum
TM: Tunicamycin
4-PBA: 4-phenyl butyric acid
CCK-8: Cell Counting Kit-8
qRT-PCR: Real-Time Polymerase Chain Reaction
elf2α: Eukaryotic initiation factor 2α
