# Supplementary figures and images for: Ascorbic acid 6-palmitate modulates microglia M1/M2 polarization in lipopolysaccharide-stimulated BV-2 cells via PERK/elF2α mediated endoplasmic reticulum stress
Source: BMC Complement Med Ther. 2022 Nov 18;22:302. doi: 10.1186/s12906-022-03780-1 (PMC9675226; doi:10.1186/s12906-022-03780-1)

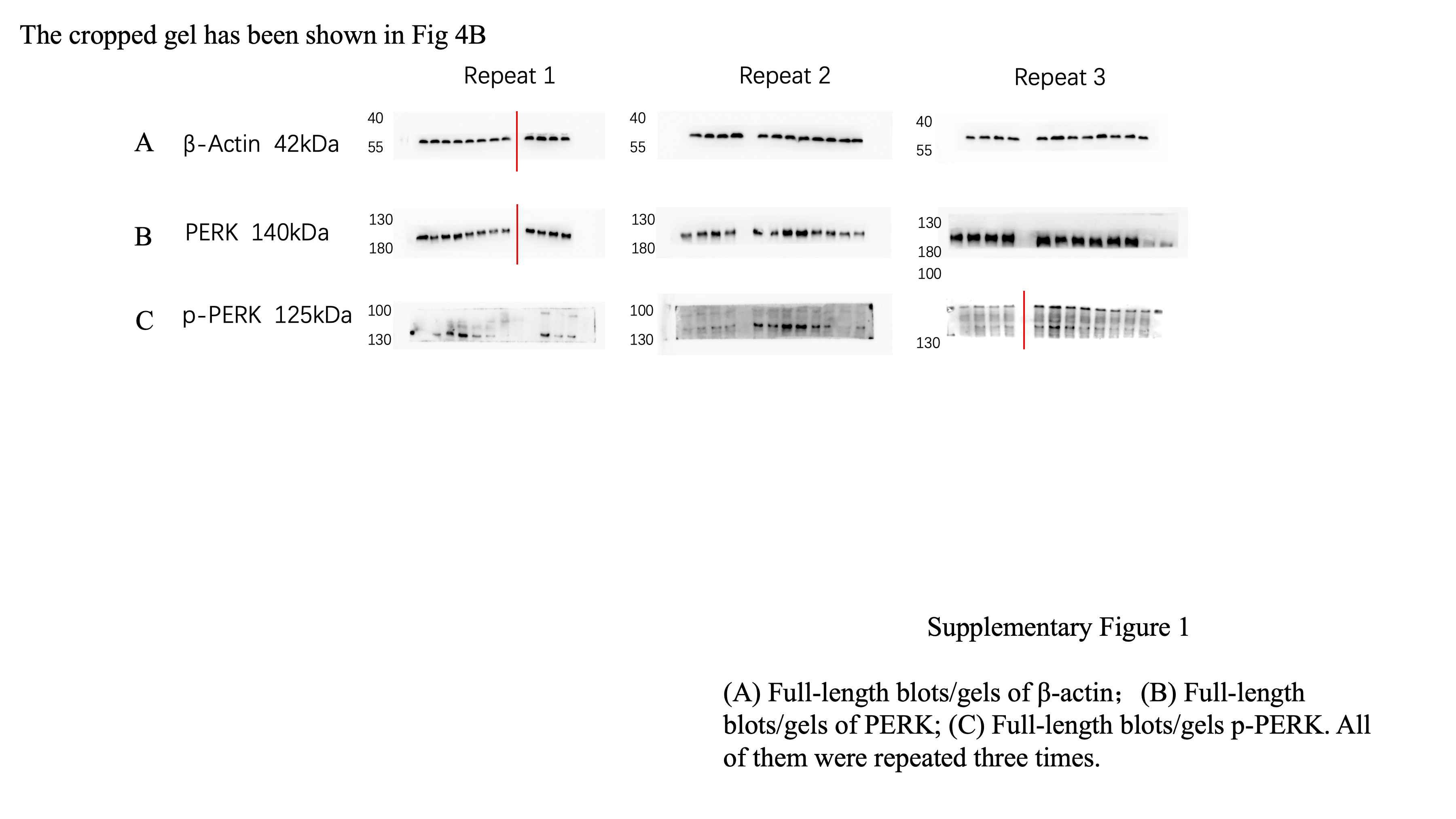

Supplement: Supplementary file 1 — Additional file 1: Supplementary figure 1. A mRNA levels of grp78 and chop in TM-stimulated BV-2 cells; B mRNA levels of inos and il-10 in TM-stimulated BV-2 cells; C mRNA levels of grp78 and chop in 4-PBA-stimulated BV-2 cells. B mRNA levels of inos and il-10 4-PBA-stimulated BV-2 cells. Supplementary figure 2. A Full-length blots/gels of β-actin; B Full-length blots/gels of PERK; C Full-length blots/gels p-PREK. All of them were repeated three times. Supplementary figure 3. Images of Full- length blots/gels of β-actin, p-PERK, and PERK, whose membrane edges can be seen. Supplementary figure 4. A Full-length blots/gels p-EIF2α; B Full-length blots/gels of β-actin. All of them were repeated three times. [file 12906_2022_3780_MOESM1_ESM.zip › Suppl Fig 1.tiff]

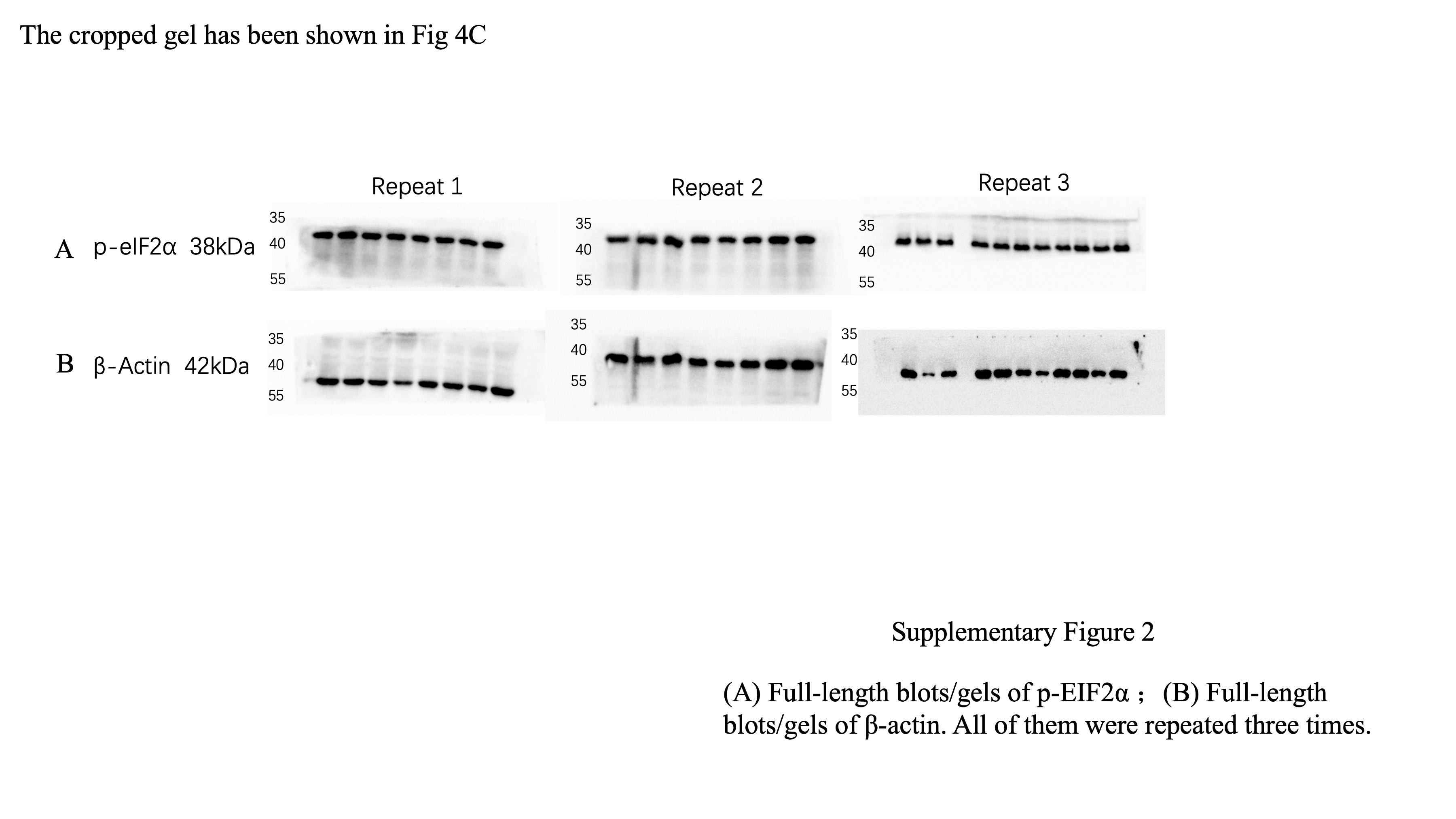

Supplement: Supplementary file 1 — Additional file 1: Supplementary figure 1. A mRNA levels of grp78 and chop in TM-stimulated BV-2 cells; B mRNA levels of inos and il-10 in TM-stimulated BV-2 cells; C mRNA levels of grp78 and chop in 4-PBA-stimulated BV-2 cells. B mRNA levels of inos and il-10 4-PBA-stimulated BV-2 cells. Supplementary figure 2. A Full-length blots/gels of β-actin; B Full-length blots/gels of PERK; C Full-length blots/gels p-PREK. All of them were repeated three times. Supplementary figure 3. Images of Full- length blots/gels of β-actin, p-PERK, and PERK, whose membrane edges can be seen. Supplementary figure 4. A Full-length blots/gels p-EIF2α; B Full-length blots/gels of β-actin. All of them were repeated three times. [file 12906_2022_3780_MOESM1_ESM.zip › Suppl Fig 2.tiff]

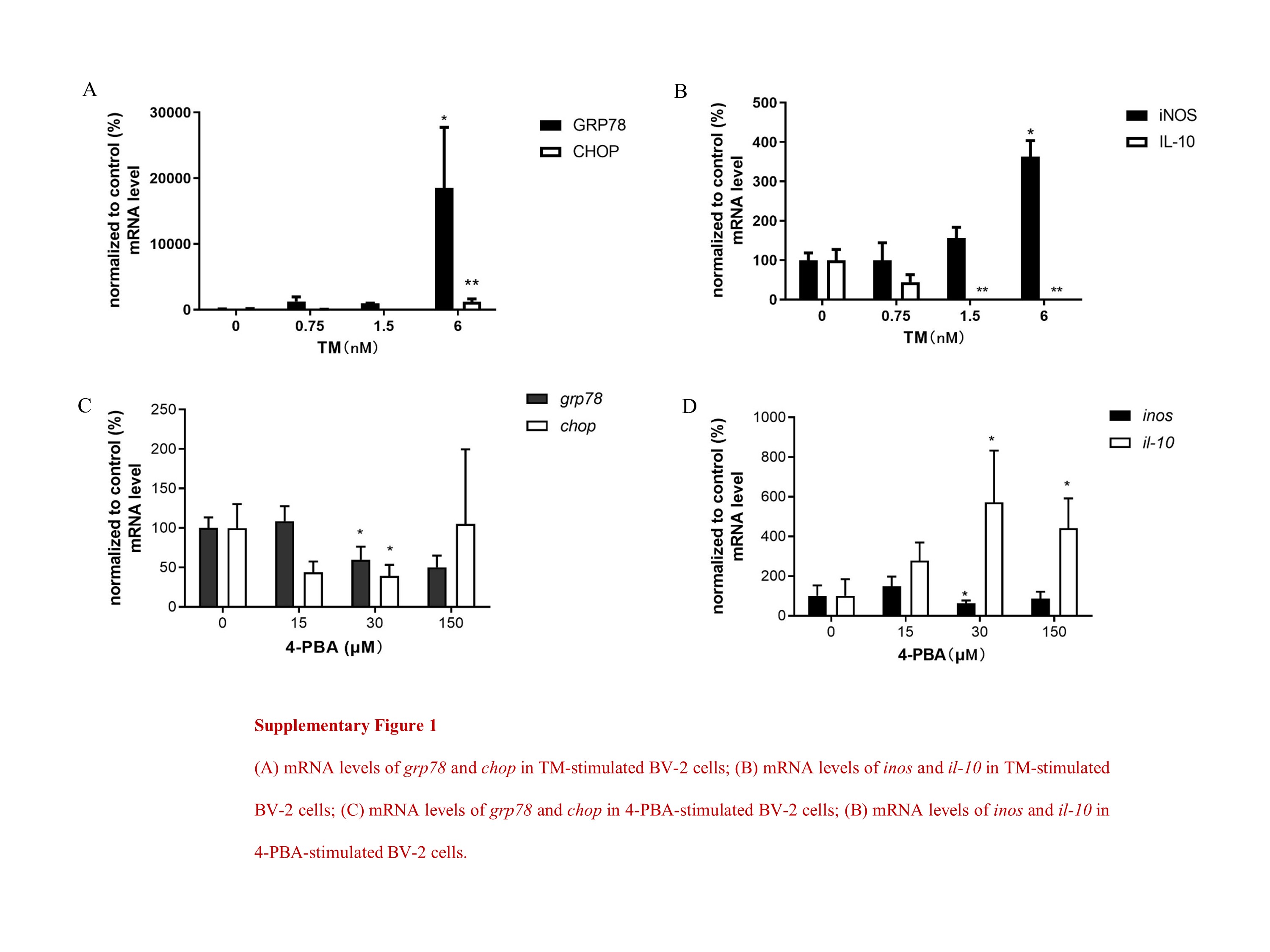

Supplement: Supplementary file 1 — Additional file 1: Supplementary figure 1. A mRNA levels of grp78 and chop in TM-stimulated BV-2 cells; B mRNA levels of inos and il-10 in TM-stimulated BV-2 cells; C mRNA levels of grp78 and chop in 4-PBA-stimulated BV-2 cells. B mRNA levels of inos and il-10 4-PBA-stimulated BV-2 cells. Supplementary figure 2. A Full-length blots/gels of β-actin; B Full-length blots/gels of PERK; C Full-length blots/gels p-PREK. All of them were repeated three times. Supplementary figure 3. Images of Full- length blots/gels of β-actin, p-PERK, and PERK, whose membrane edges can be seen. Supplementary figure 4. A Full-length blots/gels p-EIF2α; B Full-length blots/gels of β-actin. All of them were repeated three times. [file 12906_2022_3780_MOESM1_ESM.zip › Supplementary FIgure R1.JPG]

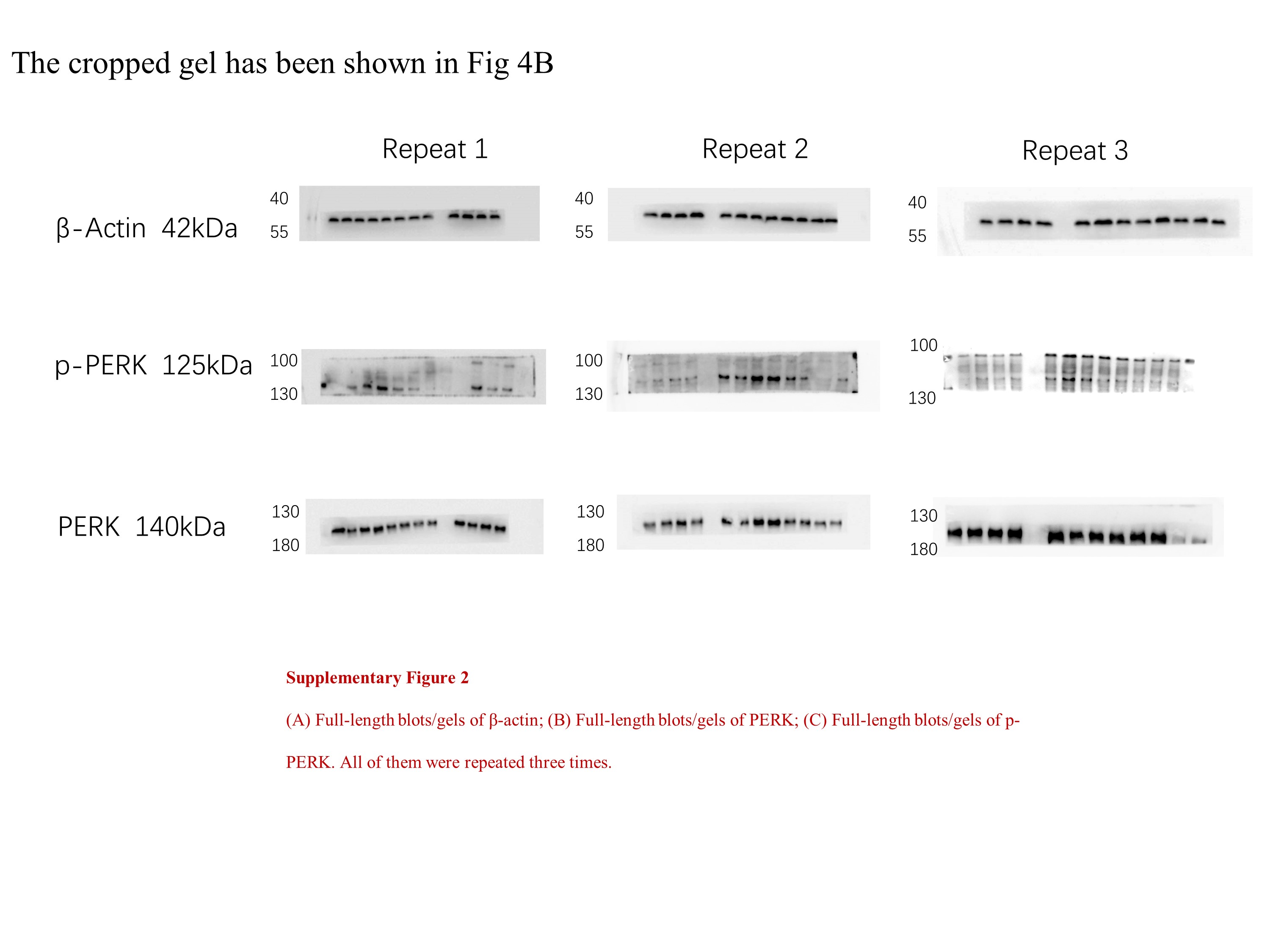

Supplement: Supplementary file 1 — Additional file 1: Supplementary figure 1. A mRNA levels of grp78 and chop in TM-stimulated BV-2 cells; B mRNA levels of inos and il-10 in TM-stimulated BV-2 cells; C mRNA levels of grp78 and chop in 4-PBA-stimulated BV-2 cells. B mRNA levels of inos and il-10 4-PBA-stimulated BV-2 cells. Supplementary figure 2. A Full-length blots/gels of β-actin; B Full-length blots/gels of PERK; C Full-length blots/gels p-PREK. All of them were repeated three times. Supplementary figure 3. Images of Full- length blots/gels of β-actin, p-PERK, and PERK, whose membrane edges can be seen. Supplementary figure 4. A Full-length blots/gels p-EIF2α; B Full-length blots/gels of β-actin. All of them were repeated three times. [file 12906_2022_3780_MOESM1_ESM.zip › Supplementary FIgure R2.JPG]

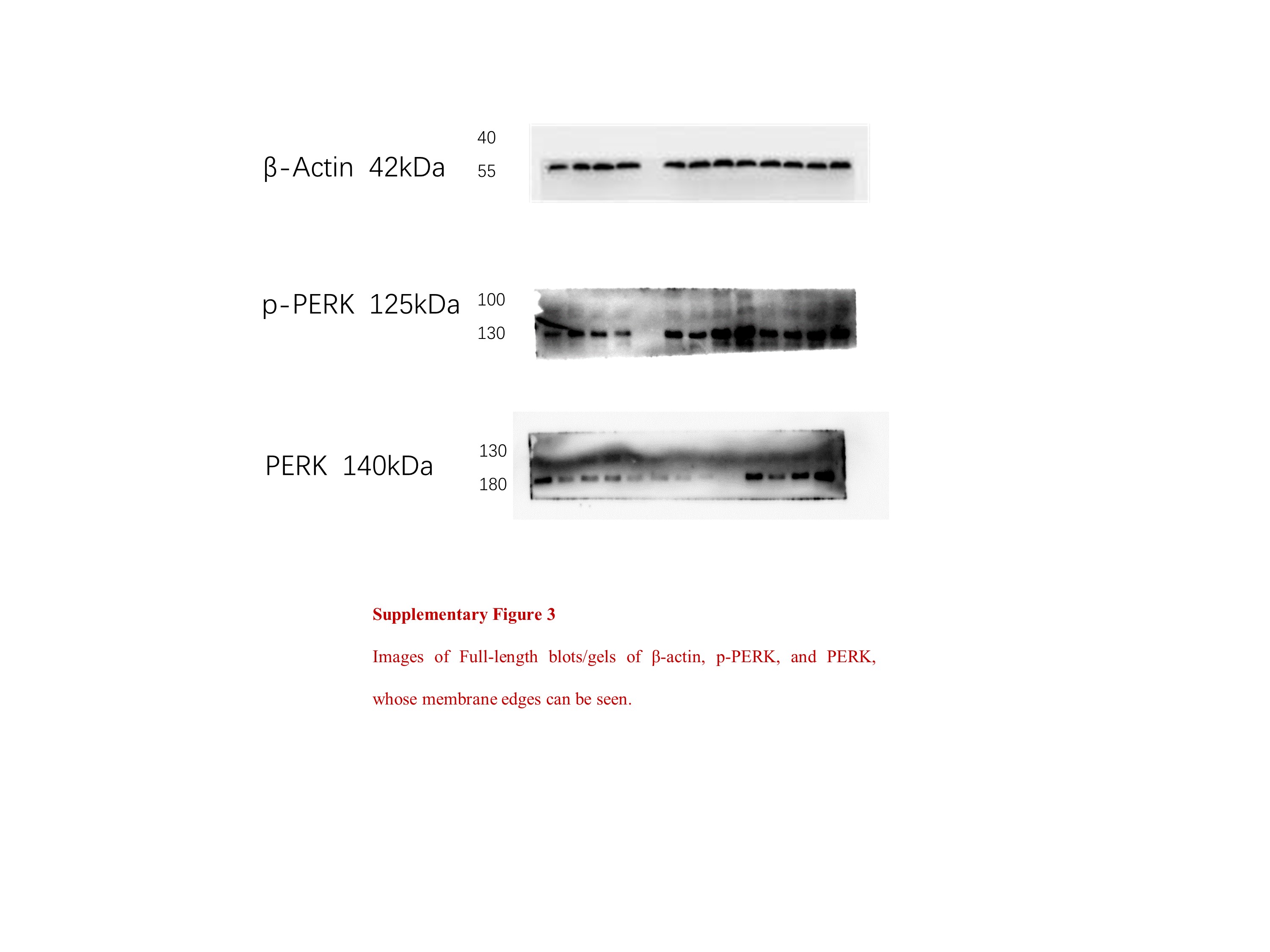

Supplement: Supplementary file 1 — Additional file 1: Supplementary figure 1. A mRNA levels of grp78 and chop in TM-stimulated BV-2 cells; B mRNA levels of inos and il-10 in TM-stimulated BV-2 cells; C mRNA levels of grp78 and chop in 4-PBA-stimulated BV-2 cells. B mRNA levels of inos and il-10 4-PBA-stimulated BV-2 cells. Supplementary figure 2. A Full-length blots/gels of β-actin; B Full-length blots/gels of PERK; C Full-length blots/gels p-PREK. All of them were repeated three times. Supplementary figure 3. Images of Full- length blots/gels of β-actin, p-PERK, and PERK, whose membrane edges can be seen. Supplementary figure 4. A Full-length blots/gels p-EIF2α; B Full-length blots/gels of β-actin. All of them were repeated three times. [file 12906_2022_3780_MOESM1_ESM.zip › Supplementary FIgure R3.JPG]

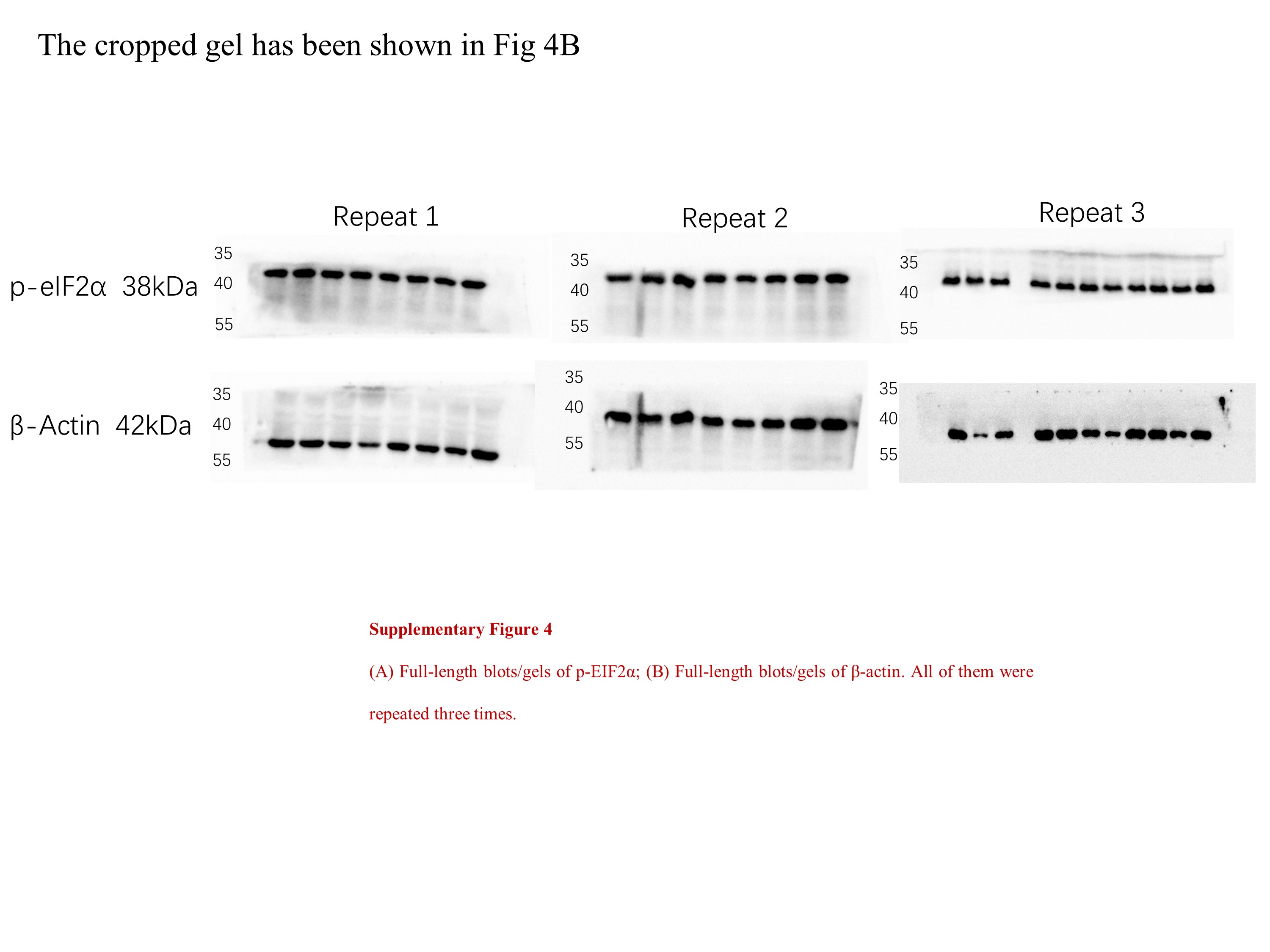

Supplement: Supplementary file 1 — Additional file 1: Supplementary figure 1. A mRNA levels of grp78 and chop in TM-stimulated BV-2 cells; B mRNA levels of inos and il-10 in TM-stimulated BV-2 cells; C mRNA levels of grp78 and chop in 4-PBA-stimulated BV-2 cells. B mRNA levels of inos and il-10 4-PBA-stimulated BV-2 cells. Supplementary figure 2. A Full-length blots/gels of β-actin; B Full-length blots/gels of PERK; C Full-length blots/gels p-PREK. All of them were repeated three times. Supplementary figure 3. Images of Full- length blots/gels of β-actin, p-PERK, and PERK, whose membrane edges can be seen. Supplementary figure 4. A Full-length blots/gels p-EIF2α; B Full-length blots/gels of β-actin. All of them were repeated three times. [file 12906_2022_3780_MOESM1_ESM.zip › Supplementary FIgure R4.JPG]
